# Supplementary material for: Automated simulation-based membrane protein refinement into cryo-EM data
Source: Biophys J. 2023 Jun 5;122(13):2773–81. doi: 10.1016/j.bpj.2023.05.033 (PMC10397807; doi:10.1016/j.bpj.2023.05.033)
Supplement: Document S1. Figures S1–S5 [file mmc1.pdf]

Biophysical Journal, Volume *122*

Supplemental information

Automated simulation-based membrane protein refinement into cryo-EM data

Linnea Yvonnesdotter, Urška Rovšnik, Christian Blau, Marie Lycksell, Rebecca Joy Howard, and Erik Lindahl

Automated simulation-based membrane-protein refinement into cryo-EM data

Linnea Yvonnesdotter[1], Urška Rovšnik[1], Christian Blau[1], Marie Lycksell[1], Rebecca J. Howard[1], and Erik Lindahl[1]

*Correspondence: erik.lindahl@scilifelab.se

SUPPORTING MATERIAL

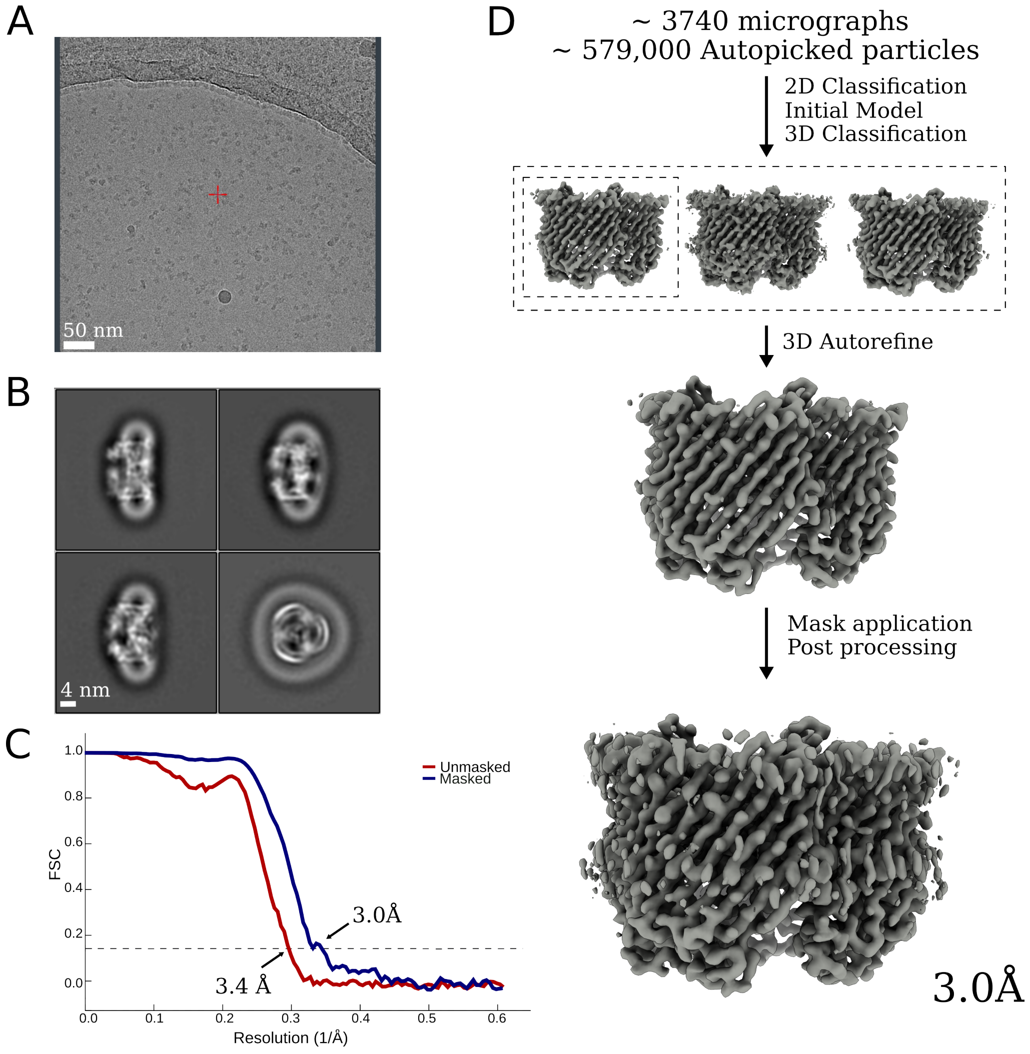

Figure S1: Maltoporin cryo-EM processing pipeline. **A** - Cryo-EM representative micrograph from the data collection. **B** - Representative 2D classes in multiple different orientations. **C** - FSC curves for unmasked (*red*) and masked (*blue*) map. **D** - Maltoporin processing pipeline with representative 3D classes, 3D refined density and a sharpened final map.

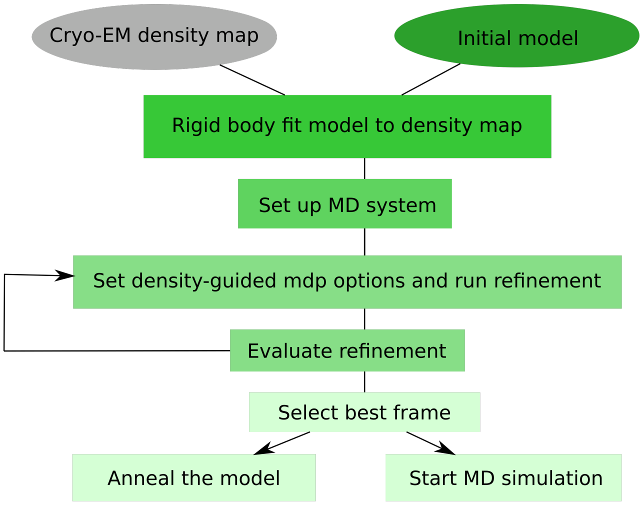

Figure S2: Suggested workflow for automated simulation-based refinement of a protein model to cryo-EM density.

Input - A cryo-EM density map or any 3D density map can be used as the target map. The initial model can for example be a model previously determined using an alternative technique or of an alternative conformation state, a homology model, etc.

Rigid body fit the model to the density map - An initial rough alignment of the initial model to the density aids in refinement. This can be done in ChimeraX using the Fit in Map tool (1). The fit protein model should then be saved relative to the target density map.

Set up MD system - The next step is to set up the molecular dynamics simulation system. For a simple solvated system GROMACS pdb2gmx, solvate, and genion functionalities can be used (2), followed by energy minimization. Membrane- or micelle-embedded systems can be set up using CHARMM-GUI bilayer builder (3) and CHARMM-GUI micelle builder respectively (4). We recommend that the size of the simulation system matches that of the target density to avoid inadvertent misalignment between map and model and checking the alignment between the target map and model, for example using VMD (5), as map and model coordinates can be interpreted differently by different tools.

Density-guided mdp options - Make sure to use the latest GROMACS version. Set the *gaussian-transform-spreading-width* according to the pixel size of the target density map as described in the methods section and more in depth by Blau et al. (6). The *density-guided-simulation-similarity-measure* affects the refinement behavior, as described by Blau et al. (6). Using relative-entropy might be advantageous for applications where a large-scale conformational change is expected or when starting from poor alignment. For refinement towards a density map containing density unaccounted for by the model relative-entropy is not recommended. We recommend *density-guided-simulation-adaptive-force-scaling* be set to true and the initial value of *density-guided-simulation-force-constant* to a low value (here we used $10e^1$ kJ/mol^{-1}). *density-guided-simulation-adaptive-force-scaling-time-constant* determines how frequently the fitting force is adapted. The default value of 4 ps has been successfully used both in this study and by Blau et al. (6), however, it might be advantageous to set the value higher for large-scale structural rearrangements, to allow for refinement during a longer period of time and thus sampling more of the conformational space.

Evaluate the refinement - We recommend that visual inspection, $FSC_{average}$, and GOAP-score be used as a minimal set of tools to evaluate the refinement. Here are some things to consider if the anticipated result has not been achieved. If a large conformational change is anticipated but not achieved it might be advantageous to refine using relative-entropy as the similarity measure, see more in (6). Another option is to increase the *density-guided-simulation-adaptive-force-scaling-time-constant*. A third option is to decrease the ruggedness of the target by fitting to a series of target maps (7). The ruggedness can be decreased by adding Gaussian noise or by downsampling the target maps. A tool for downsampling density maps is available at `https://gitlab.com/cblau/mrcsmooth/-/tree/main/`. If a part of the model is seemingly drawn outside of the density the target density map might have been extracted too narrowly. Due to the periodic nature of the simulation, it is possible that a part of the model close to the periodic boundary is refined toward the target density in an adjacent periodic box. This is more likely to happen using relative-entropy as the similarity measure as it has more long-ranged behavior, see (6). This can be solved by extracting a larger box around the density, changing the box size using the relion_image_handler tool (8), or changing the similarity measure.

Select the best frame - When satisfied with the refinement select the frame with the highest GOAP-score within the $FSC_{average}$ plateau region.

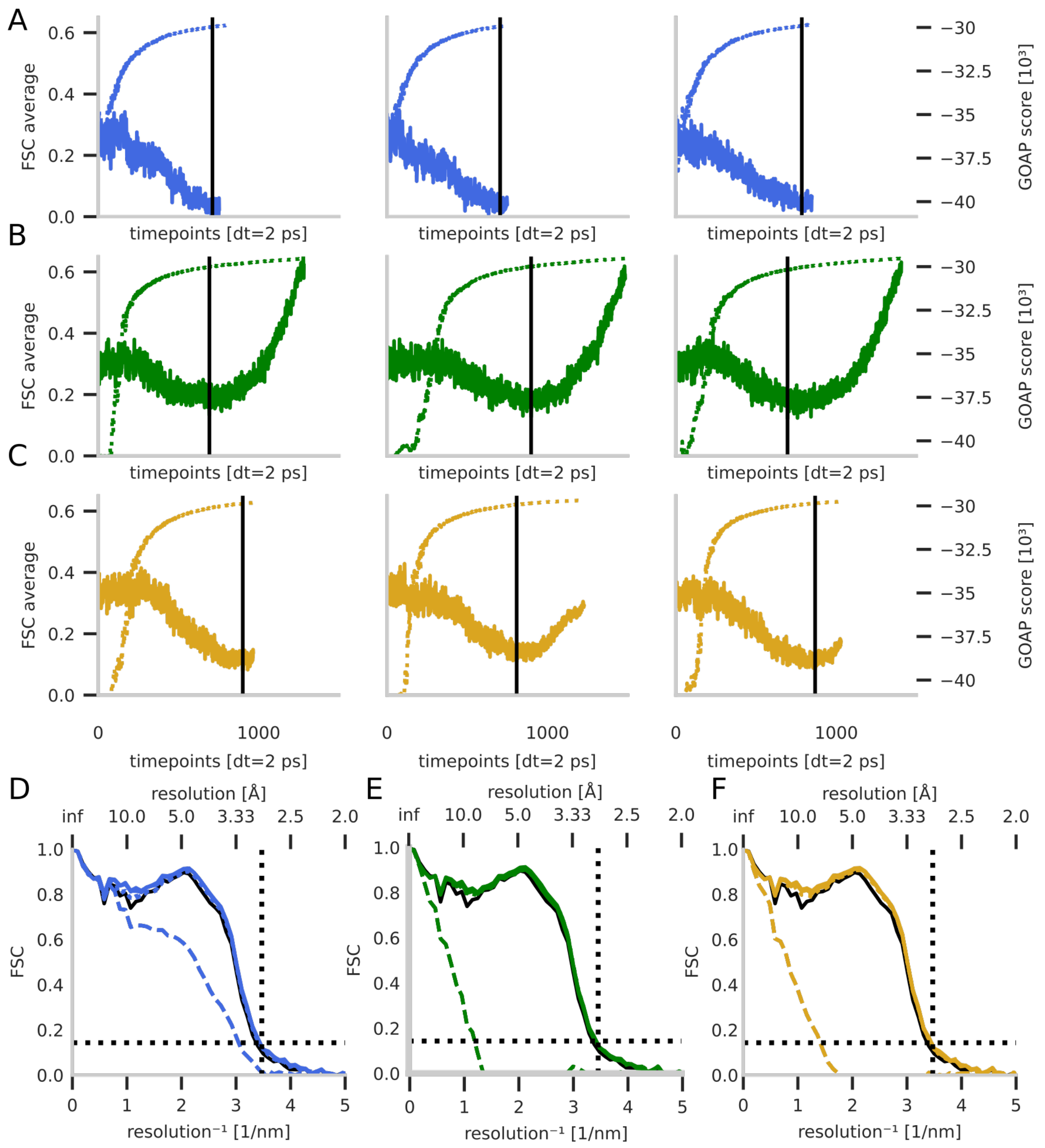

Figure S3: **A-C** - $FSC_{average}$ (dotted) and GOAP score (solid) over the refinement simulations (n=3) in solution (A), DDM (B) and POPC (C), best GOAP score frame marked with vertical black line. **D-F** - FSC of best fit position of density-guided simulations (n=3) of maltoporin (1MAL) in solution (blue), in DDM detergent micelle (green) and embedded in POPC (yellow) fit into cryo-EM density map, energy minimized models (dotted colored lines). Respective starting position marked in dashed lines and chimera fit-to-map rigid body fit starting model (solid black). Black dotted horizontal line at 0.143 and vertical at the lowest estimated local resolution of the map, 2.88 Å.

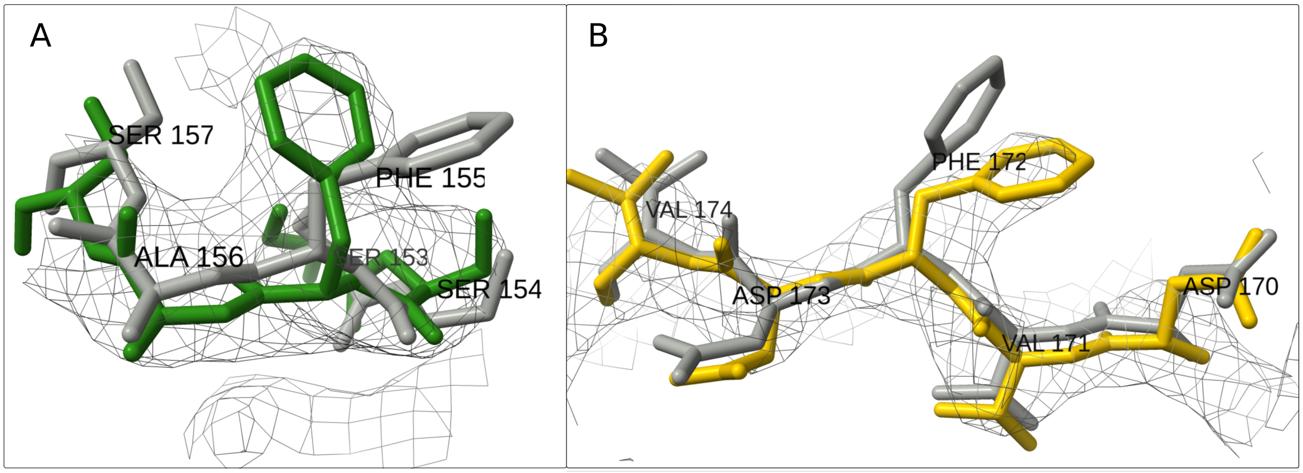

Figure S4: Local improvements in model fit to cryo-EM density after automated simulation-based refinement. **A** - Extracellular loop of maltoporin, showing the initial model (gray sticks) embedded in a DDM detergent micelle, equilibrated and rigid-body fit to the target cryo-EM density (gray mesh). Final model after density-guided simulations is shown in green. **B** - Transmembrane β-strand of maltoporin, showing initial model (gray sticks) embedded in a POPC bilayer, equilibrated and rigid-body fit to the density (gray mesh). Final model after density-guided simulations is shown in yellow.

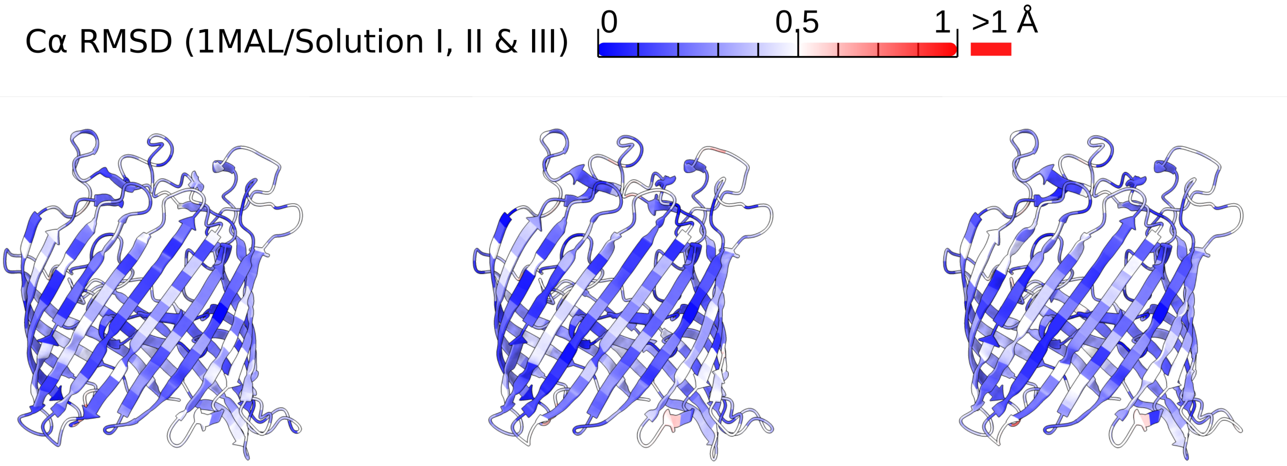

Figure S5: Moderate adjustments to secondary structure distributed throughout maltoporin after automated simulation-based refinement. Models (left—right) show final annealed state of chain A in three replicate fitting simulations (I–III) performed in solution, colored by Cα root mean-squared deviation from template X-ray structure (PDB ID 1MAL, blue=0 Å, red=1 Å).

REFERENCES

1. Pettersen, E. F., T. D. Goddard, C. C. Huang, E. C. Meng, G. S. Couch, T. I. Croll, J. H. Morris, and T. E. Ferrin, 2021. UCSF ChimeraX: Structure visualization for researchers, educators, and developers. *Protein Science* 30:70–82.

2. Páll, S., A. Zhmurov, P. Bauer, M. Abraham, M. Lundborg, A. Gray, B. Hess, and E. Lindahl, 2020. Heterogeneous parallelization and acceleration of molecular dynamics simulations in GROMACS. *The Journal of Chemical Physics* 153:134110.

3. Wu, E. L., X. Cheng, S. Jo, H. Rui, K. C. Song, E. M. Dávila-Contreras, Y. Qi, J. Lee, V. Monje-Galvan, R. M. Venable, et al., 2014. CHARMM-GUI membrane builder toward realistic biological membrane simulations.

4. Cheng, X., S. Jo, H. S. Lee, J. B. Klauda, and W. Im, 2013. CHARMM-GUI micelle builder for pure/mixed micelle and protein/micelle complex systems.

5. Humphrey, W., A. Dalke, and K. Schulten, 1996. VMD: visual molecular dynamics. *Journal of molecular graphics* 14:33–38.

6. Blau, C., L. Yvonnesdotter, and E. Lindahl, 2022. Gentle and fast all-atom model refinement to cryo-EM densities via Bayes' approach. *bioRxiv* https://www.biorxiv.org/content/early/2022/09/30/2022.09.30.510249.

7. McGreevy, R., A. Singharoy, Q. Li, J. Zhang, D. Xu, E. Perozo, and K. Schulten, 2014. xMDFF: molecular dynamics flexible fitting of low-resolution X-ray structures. *Acta Crystallographica Section D: Biological Crystallography* 70:2344–2355.

8. Nakane, T., A. Kotecha, A. Sente, G. McMullan, S. Masiulis, P. M. G. E. Brown, I. T. Grigoras, L. Malinauskaite, T. Malinauskas, J. Miehling, T. Uchański, L. Yu, D. Karia, E. V. Pechnikova, E. de Jong, J. Keizer, M. Bischoff, J. McCormack, P. Tiemeijer, S. W. Hardwick, D. Y. Chirgadze, G. Murshudov, A. R. Aricescu, and S. H. W. Scheres, 2020. Single-particle cryo-EM at atomic resolution. *Nature* 587:152 – 156.
