# Supplementary material for: Automated simulation-based membrane protein refinement into cryo-EM data
Source: Biophys J. 2023 Jun 5;122(13):2773–81. doi: 10.1016/j.bpj.2023.05.033 (PMC10397807; doi:10.1016/j.bpj.2023.05.033)
Supplement: Document S2. Article plus supporting material [file mmc2.pdf]

Biophysical Journal
Article

Biophysical Society

Automated simulation-based membrane protein refinement into cryo-EM data

Linnea Yvonnesdotter,[1] Urška Rovšnik,[1] Christian Blau,[2] Marie Lycksell,[1] Rebecca Joy Howard,[1] and Erik Lindahl[1,2,*]

[1]Science for Life Laboratory & Swedish e-Science Research Center, Department of Applied Physics, KTH Royal Institute of Technology, Solna, Sweden and [2]Science for Life Laboratory, Department of Biochemistry and Biophysics, Stockholm University, Solna, Sweden

ABSTRACT The resolution revolution has increasingly enabled single-particle cryogenic electron microscopy (cryo-EM) reconstructions of previously inaccessible systems, including membrane proteins—a category that constitutes a disproportionate share of drug targets. We present a protocol for using density-guided molecular dynamics simulations to automatically refine atomistic models into membrane protein cryo-EM maps. Using adaptive force density-guided simulations as implemented in the GROMACS molecular dynamics package, we show how automated model refinement of a membrane protein is achieved without the need to manually tune the fitting force ad hoc. We also present selection criteria to choose the best-fit model that balances stereochemistry and goodness of fit. The proposed protocol was used to refine models into a new cryo-EM density of the membrane protein maltoporin, either in a lipid bilayer or detergent micelle, and we found that results do not substantially differ from fitting in solution. Fitted structures satisfied classical model-quality metrics and improved the quality and the model-to-map correlation of the x-ray starting structure. Additionally, the density-guided fitting in combination with generalized orientation-dependent all-atom potential was used to correct the pixel-size estimation of the experimental cryo-EM density map. This work demonstrates the applicability of a straightforward automated approach to fitting membrane protein cryo-EM densities. Such computational approaches promise to facilitate rapid refinement of proteins under different conditions or with various ligands present, including targets in the highly relevant superfamily of membrane proteins.

SIGNIFICANCE Cryo-EM is an increasingly critical method of structure determination. As data collection and model generation become more efficient, iteratively fitting experimental densities still requires considerable time and expertise. Membrane proteins are particularly important targets in pharmacology and bioengineering but present distinctive challenges to data quality and modeling. Here, we tested a new tool to drive density fitting with molecular dynamics simulations, in the context of a new structure of the membrane protein maltoporin. Fitting performed well in detergent, lipids, or solution, offering simpler options for fully automated simulation protocols. We were also able to apply fitting to adjust the microscope's pixel size. The approach described here should be applicable to rapid, accurate refinement of a variety of membrane protein structures.

INTRODUCTION

Structure determination has been revolutionized by recent advances in cryogenic electron microscopy (cryo-EM). Although the speed and ease of data acquisition have increased, the interpretation of the data by model building is increasingly the bottleneck. Manual model building and refinement are time consuming and require a high level of training and expertise. Automated approaches to de novo model building show promise to speed up the process (1–3). However, there is still a need for additional refinement to improve the accuracy of models after building.

To improve the fit of a model to a density, model refinement may itself be automated by iteratively applying forces based on the experimental density to the model atoms. Additional forces, e.g., from dynamic elastic network models (4) or molecular dynamics (5,6), aim to keep the model in a reasonable biochemical state. Recent approaches to overcoming challenges in such fitting include resolution Hamiltonian exchange, varying resolution (7), force constant replica exchange (8,9), force fitting (10), and adaptive force-scaling (11). Applications of automated fitting have

Submitted October 31, 2022, and accepted for publication May 31, 2023.

*Correspondence: erik.lindahl@scilifelab.se

Editor: Michael Grabe.

https://doi.org/10.1016/j.bpj.2023.05.033

© 2023 Biophysical Society.

This is an open access article under the CC BY license (http://creativecommons.org/licenses/by/4.0/).

Check for updates

accordingly expanded from low- to higher-resolution data and from small to larger proteins (12,13). Open challenges remain in automating refinement of atomic models to cryo-EM data, particularly in the context of uncertain scaling factors and/or complex macromolecular targets.

Determining the structure of a membrane protein often involves a particular model-density discrepancy: detergents or lipids are required to solubilize the target but give rise to cryo-EM densities that are highly variable and may not directly correspond to physiological membranes. It is largely unclear how the cryo-EM density should be treated to appropriately model membrane regions in automated refinement. There are few examples available in the literature of automatic refinement of membrane proteins in a lipid environment. In work by Qi et al. (14), it is suggested that the simulation environment affects protein flexibility estimations derived from the refinement, whereas Mori et al. (15) report no effect on the result when fitting using an implicit micelle model (16) compared with implicit solvent.

In this work, we set out to determine the applicability of GROMACS density-guided simulations (11) to automatically refine a membrane protein system. Specifically, we collected and processed cryo-EM data for the *E. coli* membrane protein maltoporin, and we used a previously reported x-ray structure as a starting model to refine against the cryo-EM density in various environments. In addition to identifying a robust parameter set and model-quality metrics for refinement of membrane proteins, we report an improved estimation of the pixel size of cryo-EM data based on goodness of fit and stereochemistry of simulated models.

MATERIALS AND METHODS

Protein production and purification

Maltoporin is a protein endogenously expressed in *Escherichia coli* (17), and it co-purified with a maltose binding protein fusion construct (a ligand-gated ion channel from *Desulfofustis* deltaproteobacterium, DeCLIC (18)), which provided us with an interesting additional target to use for developing and assessing our refinement methodological work. The C43(DE3) *E. coli* cells were transformed with a pET-20b derived vector coding for DeCLIC-MBP and cultured overnight at 37°C. Cells were inoculated at 1:100 into 2xYT media with 300 μg/mL ampicillin, grown at 37°C to $OD_{600} = 0.4$. After reaching the required OD_{600}, the temperature was lowered to 20°C. The cells were further grown until the $OD_{600} = 0.8$, at which point they were induced with 100 μM isopropyl-β-D-1-thiogalactopyranoside. Membranes were harvested from cell pellets that were ultracentrifuged in a supplemented buffer A (300 mM NaCl, 20 mM Tris-HCl pH 7.6, 1.5 ku benzonase nuclease, EDTA-free protease inhibitor cocktail). Harvested cell membranes were solubilized in 2% DDM, followed by amylose affinity purification and size exclusion chromatography to isolate the fusion protein and maltoporin.

Cryo-EM sample preparation and data acquisition

Quantifoil 1.2/1.3 Cu 300 mesh grids (Quantifoil Micro Tools) were glow-discharged in the methanol vapor before sample application. 3 μL sample was applied to the grid, which was then blotted for 3 s and plunge-frozen into liquid ethane using an FEI Vitrobot Mark IV. Data collection was carried out on an FEI Titan Krios 300 kV microscope with a postenergy filter Gatan K2-Summit direct detector camera. Movies were collected at nominal 165,000x magnification, equivalent to a pixel spacing of 0.86 Å. A total dose of 42 $e^{-}/Å^{2}$ was used to collect 40 frames over 6 s, using a nominal defocus range covering −1.0 to −2.5 μm.

Image processing

All the image processing was carried out with the RELION 3.1 pipeline (19) (Fig. S1), with its own implementation of MotionCorr used for motion correction. Defocus was estimated from the motion corrected micrographs using CtfFind4 (20). After manual picking, initial 2D classification was performed to generate references for autopicking. After autopicking, particles were extracted and binned, and an initial model with C3 symmetry was generated. Particles were then aligned by performing a 3D auto-refinement. The acquired alignment parameters were used to identify and remove noise through multiple rounds of prealigned 2D and 3D classification. Micelle density was eventually subtracted, and the final 3D auto-refinement was performed using a soft mask covering the protein, followed by postprocessing with the same mask. Local resolution was estimated using the RELION implementation. Per-particle CTF parameters were estimated from the resulting reconstruction using RELION 3.1. Global beam-tilt was estimated from the micrographs, and correction was applied. The final 3D reconstruction was generated, followed by postprocessing, producing a density used for fitting. After the pixel size was calibrated, the data was reprocessed from the beginning using RELION 4.0-beta-2 (21) at 0.83 Å/px.

Automated density-guided simulations

Automated simulation-based refinement followed the workflow illustrated and described in Supporting Material (Fig. S2). The previously determined maltoporin model (PDB: 1MAL) was used as a starting conformation (17) for automated refinement. The solution system was generated in GROMACS (22) using TIP3 water and 0.15 M NaCl and energy minimized using the steepest descent algorithm that was allowed to run until convergence or a maximum force smaller than 10^{-4} kJ/mol. The CHARMM27 force field was used for all GROMACS simulations (23,24). Detergent micelle embedding of 1MAL was performed using the CHARMM-GUI micelle builder (25) in 300 DDM molecules in a 175 × 175 Å (XY) box with 25 Å water thickness. The system was solvated with TIP3 water with an ion concentration of 0.15 M NaCl. The system was equilibrated at 303.15 K within the micelle builder standard protocol. 1-palmitoyl-2-oleoyl-sn-glycero-3-phosphocholine (POPC) membrane embedding of the initial model was performed using the CHARMM-GUI bilayer builder (26) in 733 POPC molecules and solvated in TIP3 water and 0.15 M NaCl. The system was equilibrated at 303.15 K within the bilayer builder standard protocol.

Automated fitting into cryo-EM densities was performed by density-guided MD simulations using GROMACS 2021.3 (22). Density-guided simulations were performed using the refined 3D density map as a bias. Alignment of the starting model to the density map was assessed and improved iteratively using VMD (27) and the GROMACS editconf functionality, rotating and translating the structure to ensure correct global alignment before starting the simulation (22). The density-guided simulations were performed at 300 K using adaptive force scaling starting at $10e^{1}$ kJ/mol with a feedback time constant of 4 ps and a Gaussian transform spread width of the model-generated density ranging between 0.68 and 0.731 Å (2 * pixel size of target density map * 0.425 as explained by Blau et al. (11)). Map similarities were calculated from their cross correlation after normalization.

Evaluation of the overall fit of the model was performed every 2 ps using Fourier shell correlation (FSC) average values with a threshold of 2.88 Å. FSC calculations were performed using functionality available in a

modified GROMACS version, available at https://gitlab.com/gromacs/gromacs/-/commits/fscavg.

Generalized orientation-dependent all-atom potential (GOAP) score source code was downloaded at https://sites.gatech.edu/cssb/goap/ (28). GOAP scores were calculated for chain A every 2 ps and evaluated together with FSC average to find the best GOAP score within the FSC average plateau. The chosen best frame was energy minimized using the steepest descent algorithm that was allowed to run until convergence or a maximum force smaller than 10^{-4} kJ/mol/nm with the same long-range interaction settings as the density-guided molecular dynamics simulations. Phenix MolProbity scores were calculated for model quality assessment (29). FSC-Q values were calculated using the Scipion3 implementation (30,31) using default settings and a window size of 15. The generated FSC-Q maps are displayed at the default threshold as shown in ChimeraX (32).

Pixel size calibration

An initial evaluation of the pixel size was done by analyzing the radius of gyration of the fit model normalized to the maltoporin starting model (PDB: 1MAL). Values for the radius of gyration were calculated using GROMACS (22). Seven starting models were generated from an unrestrained simulation of maltoporin (PDB: 1MAL (17)). For each pixel size between 0.80 and 0.86 (0.80, 0.81, 0.82, 0.83, 0.84, 0.85, and 0.86), seven density-guided simulations were run using the cryo-EM data as target density. The GOAP score was calculated for chain A of the final frame of each simulation. The pixel size corresponding to the best average GOAP score was used as the correct pixel size estimation, and the density map was reprocessed accordingly.

DATA AVAILABILITY

Cryo-EM density maps of maltoporin in detergent micelles have been deposited in the Electron Microscopy Data Bank under accession number EMD-15903. The deposition includes the cryo-EM sharpened and unsharpened maps, both half-maps, and the mask used for final FSC calculation, as well as the RELION FSC calculated output document (21). Coordinates of the fitted model have been deposited in the Protein Data Bank, under the accession number PDB: 8B7V. The production runs, input files needed to generate a density-guided refinement, and a list of commands are available via Zenodo https://doi.org/10.5281/zenodo.7257422.

RESULTS

To build a membrane protein model in the context of a molecular dynamics force field, it is unclear how extensive the model of the protein's surrounding must be. Molecular dynamics simulations typically benefit from describing the systems in question as completely as possible, including all particles and their interactions, within the computational resources available. However, for complex systems, the setup itself is frequently nontrivial and can require extensive manual intervention, which becomes problematic in automated pipelines. Here, we first set out to test the influence of membrane mimetics of increasing complexity on density-guided simulations.

As a test system, we used cryo-EM data for a native maltoporin sample purified from *E. coli*, solubilized in the detergent n-dodecyl-β-D-maltoside (DDM). The data were processed to 3.0-Å resolution (Fig. S1; Table 1), and density corresponding to the detergent micelle was subtracted before final auto-refinement. As an initial model, we used an x-ray structure (PDB: 1MAL) previously reported at 3.1 Å (17). To test the influence of membrane mimetics, we fit the cryo-EM density with initial model systems under three different conditions of increasing complexity. In one system, the model was solvated only by water, sodium, and chloride ions, which can be performed fully automatically (Fig. 1 *A*, left). The second system had the protein embedded in a DDM micelle, using the density map before micelle subtraction to inform the number of detergent molecules needed to fill the experimental volume (Fig. 1 *A*, center). For the final system, a bilayer of POPC lipid molecules was used as a simplified cell membrane (Fig. 1 *A*, right). Each model system was run with three replicates.

To minimize potential user bias from treating a manually built model as the ground truth, we monitored quality of fit of the refined model using FSC to the target density (Fig. S3 *A–C*, dotted). All replicates of all systems achieved higher FSC to the density than did a fit-in-map rigid body fit of the starting structure performed in UCSF Chimera (33) (Fig. S3 *D–F*). The improvements were most notable in the nanometer size range, hinting at large-scale conformational improvements.

From a molecular dynamics trajectory, the aim of density fitting is to extract a single model, which balances fit to the target density with stereochemical properties. Due to the increasing forces applied during the fitting process, inherent to adaptive force scaling (11), improvement in model-to-map agreement will at some point come at the cost of stereochemical deformations. We chose the best stereochemical model, as measured by GOAP, a scoring function originally developed to assess protein structure prediction (28) (Fig. S3 *A–C*, solid). For each individual fitting simulation,

TABLE 1 Cryo-EM data collection statistics

**Data collection**	
Microscope	FEI Titan Krios
Magnification	165,000
Voltage (kV)	300
Electron exposure ($e^-/Å^2$)	~ 40
Defocus range (μm)	−2.0 to −3.6
Pixel size (Å)	0.83
Symmetry imposed	C3
Number of images	3740
Particles picked	~ 579,000
Particles refined	37,521
**Refinement**	
Resolution (Å)	3.0
FSC threshold	0.143
Map sharpening B-factor	−70

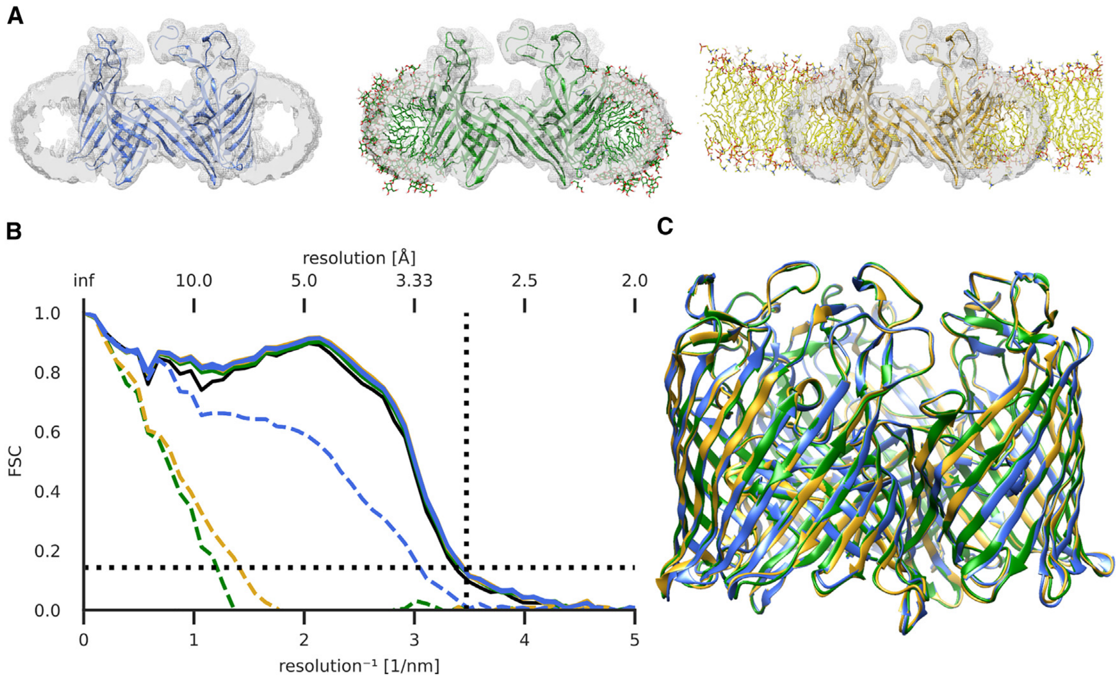

FIGURE 1 Effect of membrane mimetic on the quality of model fitting to detergent-solubilized maltoporin cryo-EM density. (*A*) Cryo-EM density map (gray) before micelle subtraction, superposed with an initial model of maltoporin (PDB: 1MAL) in solution (*left*), DDM detergent micelle (*middle*), or POPC lipid bilayer (*right*). (*B*) FSC of the best-fit model from density-guided simulations ($n = 3$) of maltoporin in solution (*blue*), detergent (*green*), or lipid (*yellow*) fit into the same cryo-EM density map. FSC of initial models marked in dashed lines and Chimera fit-in-map rigid body fit model (*solid black*). Black dashed horizontal line at 0.143 and vertical at the lowest estimated local resolution of the map, 2.88 Å. (*C*) Overlay of fitted models. To see this figure in color, go online.

the lowest (i.e., best) GOAP score corresponded to a point within the FSC $_{average}$ plateau, close to the maximum value. All simulations terminated within 3 ns due to excess adapted forces applied. Although models fit in solution (Fig. S3 *A*) achieved better GOAP scores than those in detergent (Fig. S3 *B*) or lipid (Fig. S3 *C*), the best-fit models were effectively superimposable (Fig. 1 *C*). Moreover, despite starting at different offsets compared with the target density map, there was no clear difference in best-fit model-to-map FSC between the three membrane-mimetic systems (Figs. 1 *B* and S3 *D–F*). As a final test of the stereochemical quality of our models and the influence of the membrane mimetic, we used MolProbity scores (29) and found improvement relative to the initial model in all conditions, with the best scores observed for models refined in solution (Table 2). The best-fit simulation frame from each replicate was energy minimized with heavy-atom restraints, resulting in final annealed models with even better MolProbity scores (Table 3). A workflow for the proposed automated fitting approach is illustrated and described in Supporting Material, including suggestions for parameter tuning (Fig. S2).

Automated simulation-based refinement visibly improved the fit to cryo-EM data of amino acid side chains from models equilibrated in multiple solvation conditions (Fig. S4), as well as inducing moderate adjustments to secondary structure in both the transmembrane β-strands and soluble loops (Fig. S5). To ensure that we made full use of the structural information while avoiding over-fitting, we further evaluated our structures in atomic detail using FSC-Q (30). This quality measure compares model-to-map differences with differences between half-maps and was recently integrated into the cryo-EM data processing framework Scipion3 (31). Values of FSC-Q more negative than -0.5 indicate that an atom may have been refined to noise, whereas values above $+0.5$ indicate low correlation with the density or low resolution of the density. Best-fit models refined in solution, detergent, or lipid had similar mean FSC-Q scores between 0.12 and 0.17 (Fig. 2). Solution models had a higher percentage of atoms below -0.5 and above $+0.5$ than those in detergent or lipid; still, for all systems, more than 93% of atoms had FSC-Q values in the well-fitted (-0.5 to $+0.5$) range.

Interestingly, density-guided simulations provided an unanticipated opportunity to refine a parameter of our original cryo-EM reconstruction. Specifically, we sought to validate the pixel size estimate of our micrographs, reported at the time of data collection as 0.86 Å/pixel. For pixel size estimates that are too large, we expect an overall stretching of the model, whereas for too small estimates, we expect a compression. We refined the pixel size estimate with two approaches. First, we compared the overall shape of the model to a reference structure, the initial model (PDB: 1MAL (17)), during density-guided simulation, using radius of gyration. Due to the anisotropy of membrane systems, we

TABLE 2 Model quality statistics at the best-fit frame of the density-guided simulation trajectories (n = 3) in solution, DDM, and POPC

Replicate	Ramachandran outliers (%)	Favored (%)	Rotamer outliers (%)	C-β deviations	Clashscore	RMS (bonds)	RMS (angles)	MolProbity score
Solution I	0.56	94.43	1.77	83.0	0.47	0.372	3.39	1.23
Solution II	0.48	94.51	2.39	73.0	0.32	0.0379	3.41	1.28
Solution III	0.72	94.27	2.08	87.0	0.63	0.0403	3.46	1.34
DDM I	0.91	92.44	3.6	91.0	0.16	0.033	3.45	1.46
DDM II	1.0	93.43	3.39	109.0	0.95	0.033	3.45	1.62
DDM III	0.75	93.85	3.7	111.0	0.64	0.0329	3.44	1.55
POPC I	0.42	93.85	3.17	82.0	0.37	0.04	3.28	1.43
POPC II	0.91	93.77	3.49	85.0	0.37	0.0383	3.37	1.46
POPC III	0.67	93.02	3.28	90.0	0.05	0.0417	3.47	1.36
1MAL	0.48	91.41	15.29	27	19.68	0.0201	2.19	3.20

calculated independently the radii of gyration around the membrane normal and two axes perpendicular to it, normalized to those of the reference structure. When fit to the original cryo-EM map processed at 0.86 Å/pixel, refinements converged to ~3% larger radius of gyration than the reference structure (Fig. 3 *A*), suggesting that 0.86 Å/pixel may be larger than the actual pixel size during data collection.

To estimate the correct pixel size, we rescaled the cryo-EM map to voxel sizes ranging from 0.80 to 0.85 Å. Using a modified version of the approach suggested by Tiwari et al. (34), structures at seven different timepoints of an unrestrained molecular dynamics simulation of the initial model (PDB ID: 1MAL (17)) were used as seeds for density-guided refinement into the six rescaled densities and the initial cryo-EM density (resulting in $7\,x\,7 = 49$ fitting simulations). As any gross geometrical change is expected to deteriorate the overall stereochemical quality of the models, we used the average GOAP score to select the most suitable pixel size. This approach thus avoids reliance on a reference structure for the fitted model. Fitting to rescaled maps improved the GOAP scores of best-fit models, with optimal scores at 0.83 Å/pixel (Fig. 3 *B*). The best-fit model refined at 0.83 Å/pixel converged to within 1% of the radius of gyration of the initial structure, again with relative contraction along the z axis (Fig. 3 *C*). Pixel-size estimation is an ongoing concern of independent and facility cryo-EM users (35), often accomplished only by collecting and calibrating against high-resolution data from a standard sample such as apoferritin (36). After determining our modified pixel-size estimate by computational means, subsequent discussions with the cryo-EM facility were consistent with a need to revise the estimate for our data set, as well as others collected in that period of time. Accordingly, all analyses in this work (except Fig. 3) correspond to cryo-EM data reprocessed from original micrographs at 0.83 Å/pixel.

DISCUSSION

Despite the recent rapid increase in cryo-EM structure depositions, membrane protein structures remain underrepresented, particularly relative to their importance to physiological signaling and drug development. As data collection becomes increasingly efficient, automated modeling of atom coordinates into a 3D map is poised to become a critical tool for interpreting cryo-EM data and as a starting point for further molecular dynamics simulations. Here, we tested the applicability of a recently reported density-guided fitting tool (11) in the GROMACS software suite to membrane proteins and their membrane mimetics, including a proposed workflow (Fig. S2). We used new cryo-EM data for maltoporin, representative of a large class of β-barrel transmembrane proteins (17,37,38). We predicted that maltoporin's relative rigidity would avoid large-scale conformational transitions confounding the

TABLE 3 Model quality statistics of best-fit frame of the density-guided simulation trajectories (n = 3) after heavy-atom-restrained energy minimization in solution, POPC, and DDM

Replicate	Ramachandran outliers (%)	Favored (%)	Rotamer outliers (%)	C-β deviations	Clashscore	RMS (bonds)	RMS (angles)	MolProbity score
Solution I	0.64	96.74	1.87	8.0	0.00	0.0154	1.92	0.91
Solution II	0.80	96.26	2.80	8.0	0.05	0.0154	1.92	1.11
Solution III	0.56	95.78	2.18	6.0	0.11	0.0154	1.93	1.09
DDM I	0.42	95.59	2.33	9.0	0.05	0.0152	1.89	1.11
DDM II	0.67	94.68	1.90	9.0	0.00	0.0153	1.89	1.08
DDM III	0.58	95.26	2.33	7.0	0.05	0.0153	1.90	1.13
POPC I	0.33	96.09	2.01	7.0	0.05	0.0152	1.88	1.02
POPC II	0.58	95.68	1.90	10.0	0.05	0.0153	1.89	1.03
POPC III	0.50	96.18	2.22	11.0	0.05	0.0153	1.89	1.05
1MAL	0.48	91.41	15.29	27.0	19.68	0.0201	2.19	3.20

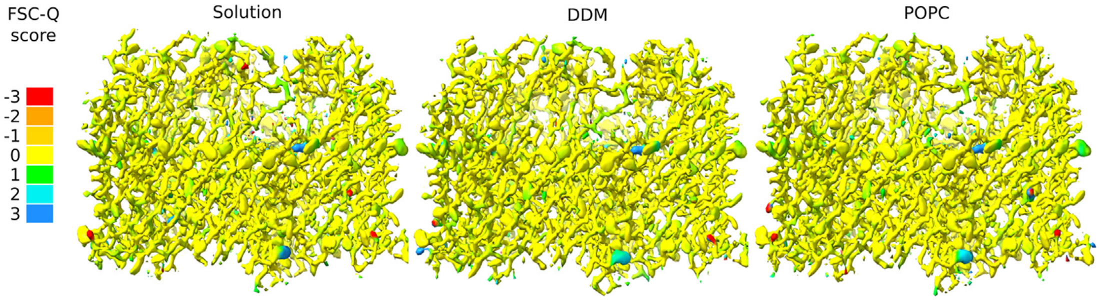

Replicate		I	II	III	I	II	III	I	II	III
Mean FSC-Q		0.13	0.17	0.17	0.12	0.12	0.16	0.12	0.17	0.12
%FSC-Q	> 0.5	4.64	5.32	5.48	4.38	3.96	5.47	3.80	4.14	3.56
	<-0.5	1.10	1.19	1.21	0.74	0.73	1.06	0.61	0.70	0.64

FIGURE 2 FSC-Q quality validation of models fit in solution (*left*), detergent (*middle*), or lipid (*right*). Densities generated from fit models, colored by residue FSC-Q values (>0.5 indicating poor correlation or low resolution of the density map, and <–0.5 indicating potential over-fitting). Mean FSC-Q values and percentage of atoms with values >0.5 and <–0.5 are indicated for three replicates (I–III) for each simulation setup. To see this figure in color, go online.

fitting dynamics and would enable precise assessment of membrane-mimetic effects by minimizing restructuring of lipidic components around mobile hydrophobic regions.

As implemented here, the conditions chosen to mimic the embedding membrane—aqueous solution, detergent micelle, or lipid bilayer—did not substantially influence the overall FSC between map and best-fit model. This agnosticism is consistent with previous indications of Mori et al., who achieved high-quality automated refinement using a different tool even in implicit solvent (15). We attribute this seemingly surprising success of modeling a membrane protein in the absence of a solubilizing environment to several factors. To a lesser extent, we expect density-guided simulations to induce slightly hydrophobic behavior by reducing side-chain flexibility and thus the number of possible hydrogen bonding configurations (39). We attribute a larger role to the forces from the density fitting, where an adaptive force constant adjusts the bias of the target density to overpower the influence of the embedding environment.

The GROMACS-embedded tool implemented in this work (11) is one of a handful currently available for automated fitting of cryo-EM structures. The adaptive force constant described above is similar to the replica exchange force constant in GENESIS (8), whereas it is different to the fixed force constant employed in MDFF (14). Otherwise, MDFF is likely the most similar protocol to that described here; however, our approach does not require setting the force constant or using additional secondary structure constraints, as described in detail in Blau et al. (11). The ISOLDE (40) implementation of MDFF as a plugin to ChimeraX (32) allows for interactive user intervention, and with that come some necessary approximations to the size and thoroughness of the force field description. Whereas the approach described here takes the whole system including solvent into account, ISOLDE aids users to focus on select regions that need further refinement. It is in this sense orthogonal to our approach as it requires, but also allows, manual interaction in specific regions. The whole-system treatment offers an advantage in our case, as we can take membrane molecules and their rearrangement into account. However, there might be instances where the initial description of the model contains serious errors. This may require manual re-modeling by a skilled user, either interactively as done in ISOLDE or before our automated approach. A possible scenario would be to run our fully automated refinement first and then follow up with interactive refinement in specific regions if need be.

The map-to-model quality measured by FSC-Q (30) was also comparable for all membrane-mimetic systems tested. We did however observe a slightly lower percentage of atoms with FSC-Q values below -0.5, indicating reduced over-fitting, in the detergent- and lipid-embedded models than in solution. We theorize that embedding in detergent or lipid might reduce fitting to noise left in a density map after micelle subtraction by physically blocking that space. For density maps with high levels of noise, or with lipid or other density, not associated with the target protein, it might be advantageous to refine the protein in an embedded system; however, with well-defined density or tight mask, the difference should be small.

Models refined in solution achieved better GOAP scores than their detergent- or lipid-embedded counterparts. One contributing factor to this may be that GOAP is a knowledge-based score based on a set of 1011 nonhomologous proteins with a resolution of <2 Å available in the PDB (41). As membrane proteins remain underrepresented in the PDB, GOAP score may represent the characteristics of proteins in solution better than a protein embedded in detergent or a lipid bilayer.

Although most cryo-EM facilities perform regular pixel size calibrations to ensure correct scaling, there is sometimes a need for re-estimation. The change in radius of gyration to a reference structure has proven useful to give a

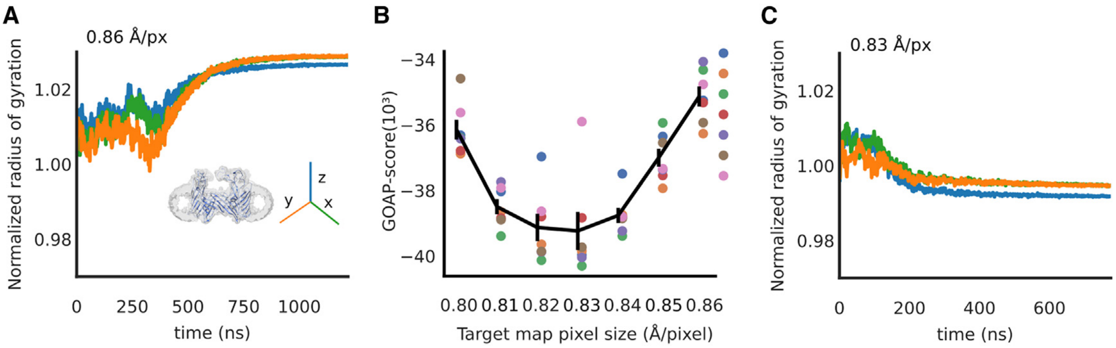

FIGURE 3 Pixel-size estimation improved by density-guided simulations. (*A*) Radius of gyration of a fitted model, normalized to that of the initial model (PDB: 1MAL), guided by the experimental density map processed at the pixel size estimated at time of data collection (0.86 Å/pixel). Dimensions of the protein are plotted separately along x (*green*), y (*orange*), and z (*blue*) axes, the latter perpendicular to the membrane plane. (*B*) Optimal GOAP scores for density-guided simulations initiated from a range of starting models (n = 7, colored separately) guided by maps scaled at 0.80–0.86 Å/pixel. Solid line represents mean ± standard error of GOAP scores from fitting runs at each pixel size. (*C*) Radius of gyration as in (*A*) for one model fitted to a map scaled at the optimized pixel size (0.83 Å/pixel). To see this figure in color, go online.

first indication at the scale and direction of the error. A caveat to this approach is that it requires a known reference structure in the same conformational state as the target density. Further, taking an x-ray model as the ground truth for the overall shape of a protein is potentially problematic, as we lose the ability to find cryo-EM specific characteristics, which might stem from the difference in temperature at which structures are solved or from the absence of crystal contacts. Instead, density-guided fitting in combination with GOAP scoring (28) can be used for such re-estimation, similar to the methodology proposed by Tiwari et al. (34). This approach scores the soundness of the model stereochemistry, rather than comparing to a single reference. Again, as the GOAP score itself is based on a heuristic from a set of structures, mainly solved by x-ray crystallography (41), it might affect the results, as we compare between data derived from x-ray and cryo-EM. Finally, in order to deposit a final model to PDB. it is necessary to re-process the data with the found correct pixel size, as no additional processing, such as re-scaling, of half-maps is allowed outside of the EM-processing tool for PDB deposition.

Adaptive force scaling allows sampling at different balances between stereochemistry and goodness of fit. Given that the cryo-EM density represents a thermodynamically favorable state, we expect that there is a corresponding minimum in the GOAP score of a correctly fit model to that density. In this case of maltoporin, devoid of large conformational changes, we find one GOAP score minimum at the FSC plateau. However, it should be possible to see two minima separated by a barrier when fitting between conformational states, one corresponding to the initial conformation and the other to the conformation represented by the target density. The protocol suggested here for automatically refining membrane proteins has been optimized for a relatively rigid protein. Except for the pixel size-dependent spread width, fitting parameters were equivalent to those used to fit a variety of soluble protein models in other recent work (11), indicating these defaults are widely applicable. Still, it is possible that some tweaking of the parameter set could aid in refining a more flexible protein. We then suggest increasing the time constant τ that regulates the interval at which the adaptive force constant is increased or decreased, allowing for more sampling of the conformational space at each increment of the added bias. For large conformational changes, we would expect an approach using density maps of increasing resolution, as reported by McGreevy et al. (42), combined with adaptive force scaling, to be a viable strategy. Such adaptations may also be applicable to the use of alternative starting models, particularly from structure prediction tools such as AlphaFold2 (43), which promise testable models for a range of less accessible proteins, but whose precision and accuracy remain unclear. Here we show how density-guided simulations can be used to refine membrane protein modeling; the same approach should be applicable to fitting predicted structures, lipids, or ligands into experimental data.

CONCLUSION

In summary, we tested here an approach to automated model refinement of membrane proteins using density-guided simulations in GROMACS, consisting of the following steps: rigid-body alignment of an initial model to the density; setting up a solution-phase MD simulation; monitoring $FSC_{average}$ and GOAP scores during density-guided simulations; selecting the simulation snapshot with the best GOAP score that falls on the $FSC_{average}$ plateau; energy-minimizing the selected snapshot with heavy atoms restrained; and validating the final model on the basis of FSC, FSC-Q, GOAP score, and MolProbity score. Parallel simulations with variously scaled densities may also be useful in optimizing pixel-size estimation.

SUPPORTING MATERIAL

Supporting material can be found online at https://doi.org/10.1016/j.bpj.2023.05.033.

AUTHOR CONTRIBUTIONS

Conceptualization: L.Y., U.R., and C.B.; methodology: L.Y. and C.B.; software: L.Y., C.B., and U.R.; validation: L.Y.; formal analysis: L.Y., U.R., M.L., C.B., R.H., and E.L.; investigation: L.Y., U.R., C.B., and M.L.; data curation: L.Y. and U.R.; original draft: L.Y.; review and editing: L.Y., U.R., M.L., C.B., R.H., and E.L.; visualization: L.Y. and U.R.; supervision: R.J.H. and E.L.; project administration: R.J.H. and E.L.; funding acquisition: E.L.

ACKNOWLEDGMENTS

The authors would like to thank the Swedish Cryo-EM National Facility staff, especially Marta Carroni and Stefan Fleischmann from Stockholm and Michael Hall from Umeå, for their kind assistance with data collection. The Facility is funded by the Knut and Alice Wallenberg Foundation, Erling Persson and Kempe Foundations. This project was supported by the Swedish Research Council (2019-02433, 2021-05806), the Swedish e-Science Research Centre, and the BioExcel Center of Excellence (EU-823830). Computational resources were provided by the Swedish National Infrastructure for Computing (SNIC).

DECLARATION OF INTERESTS

The authors declare no competing interests.

REFERENCES

1. Terwilliger, T. C., P. D. Adams, …, O. V. Sobolev. 2018. A fully automatic method yielding initial models from high-resolution cryo-electron microscopy maps. *Nat. Methods.* 15:905–908.
2. Pfab, J., N. M. Phan, and D. Si. 2021. DeepTracer for fast de novo cryo-EM protein structure modeling and special studies on CoV-related complexes. *Proc. Natl. Acad. Sci. USA.* 118, e2017525118.
3. Terashi, G., and D. Kihara. 2018. De novo main-chain modeling for EM maps using MAINMAST. *Nat. Commun.* 9:1618–1711.
4. Wang, Z., and G. F. Schröder. 2012. Real-space refinement with DireX: from global fitting to side-chain improvements. *Biopolymers.* 97:687–697.
5. Trabuco, L. G., E. Villa, …, K. Schulten. 2008. Flexible fitting of atomic structures into electron microscopy maps using molecular dynamics. *Structure.* 16:673–683.
6. Orzechowski, M., and F. Tama. 2008. Flexible fitting of high-resolution x-ray structures into cryoelectron microscopy maps using biased molecular dynamics simulations. *Biophys. J.* 95:5692–5705.
7. Singharoy, A., I. Teo, …, K. Schulten. 2016. Molecular dynamics-based refinement and validation for sub-5 Å cryo-electron microscopy maps. *Elife.* 5, e16105.
8. Miyashita, O., C. Kobayashi, …, F. Tama. 2017. Flexible fitting to cryo-EM density map using ensemble molecular dynamics simulations. *J. Comput. Chem.* 38:1447–1461.
9. Kulik, M., T. Mori, and Y. Sugita. 2021. Multi-scale flexible fitting of proteins to cryo-EM density maps at medium resolution. *Front. Mol. Biosci.* 8, 631854.
10. Igaev, M., C. Kutzner, …, H. Grubmüller. 2019. Automated cryo-EM structure refinement using correlation-driven molecular dynamics. *Elife.* 8, e43542.
11. Blau, C., L. Yvonnesdotter, and E. Lindahl. 2022. Gentle and fast all-atom model refinement to cryo-EM densities via Bayes' approach. Preprint at bioRxiv. https://doi.org/10.1101/2022.09.30.510249.
12. Nakane, T., A. Kotecha, …, S. H. W. Scheres. 2020. Single-particle cryo-EM at atomic resolution. *Nature.* 587:152–156.
13. Yip, K. M., N. Fischer, …, H. Stark. 2020. Atomic-resolution protein structure determination by cryo-EM. *Nature.* 587:157–161.
14. Qi, Y., J. Lee, …, W. Im. 2017. CHARMM-GUI MDFF/xMDFF utilizer for molecular dynamics flexible fitting simulations in various environments. *J. Phys. Chem. B.* 121:3718–3723.
15. Mori, T., G. Terashi, …, Y. Sugita. 2021. Efficient flexible fitting refinement with automatic error fixing for de novo structure modeling from cryo-EM density maps. *J. Chem. Inf. Model.* 61:3516–3528.
16. Mori, T., and Y. Sugita. 2020. Implicit micelle model for membrane proteins using ellipsoid approximation. *J. Chem. Theor. Comput.* 16:711–724.
17. Schirmer, T., T. A. Keller, …, J. P. Rosenbusch. 1995. Structural basis for sugar translocation through maltoporin channels at 3.1 Å resolution. *Science.* 267:512–514.
18. Hu, H., R. J. Howard, …, M. Delarue. 2020. Structural basis for allosteric transitions of a multidomain pentameric ligand-gated ion channel. *Proc. Natl. Acad. Sci. USA.* 117:13437–13446.
19. Zivanov, J., T. Nakane, and S. H. W. Scheres. 2020. Estimation of high-order aberrations and anisotropic magnification from cryo-EM data sets in RELION-3.1. *IUCrJ.* 7:253–267.
20. Rohou, A., and N. Grigorieff. 2015. CTFFIND4: fast and accurate defocus estimation from electron micrographs. *J. Struct. Biol.* 192:216–221.
21. Kimanius, D., L. Dong, …, S. H. W. Scheres. 2021. New tools for automated cryo-EM single-particle analysis in RELION-4.0. *Biochem. J.* 478:4169–4185.
22. Abraham, M. J., T. Murtola, …, E. Lindahl. 2015. GROMACS: high performance molecular simulations through multi-level parallelism from laptops to supercomputers. *SoftwareX.* 1-2:19–25.
23. Mackerell, A. D., Jr., M. Feig, and C. L. Brooks, III. 2004. Extending the treatment of backbone energetics in protein force fields: limitations of gas-phase quantum mechanics in reproducing protein conformational distributions in molecular dynamics simulations. *J. Comput. Chem.* 25:1400–1415.
24. Bjelkmar, P., P. Larsson, …, E. Lindahl. 2010. Implementation of the CHARMM force field in GROMACS: analysis of protein stability effects from correction maps, virtual interaction sites, and water models. *J. Chem. Theor. Comput.* 6:459–466.
25. Cheng, X., S. Jo, …, W. Im. 2013. CHARMM-GUI micelle builder for pure/mixed micelle and protein/micelle complex systems. *J. Chem. Inf. Model.* 53:2171–2180.
26. Wu, E. L., X. Cheng, …, W. Im. 2014. CHARMM-GUI membrane builder toward realistic biological membrane simulations. *J. Comput. Chem.* 35:1997–2004.
27. Humphrey, W., A. Dalke, and K. Schulten. 1996. VMD: visual molecular dynamics. *J. Mol. Graph.* 14:33–38.
28. Zhou, H., and J. Skolnick. 2011. GOAP: a generalized orientation-dependent, all-atom statistical potential for protein structure prediction. *Biophys. J.* 101:2043–2052.
29. Williams, C. J., J. J. Headd, …, D. C. Richardson. 2018. MolProbity: more and better reference data for improved all-atom structure validation. *Protein Sci.* 27:293–315.
30. Ramírez-Aportela, E., D. Maluenda, …, C. O. S. Sorzano. 2021. FSC-Q: a CryoEM map-to-atomic model quality validation based on the local Fourier shell correlation. *Nat. Commun.* 12:1–7.
31. de la Rosa-Trevín, J. M., A. Quintana, …, J. M. Carazo. 2016. Scipion: a software framework toward integration, reproducibility and validation in 3D electron microscopy. *J. Struct. Biol.* 195:93–99.
32. Pettersen, E. F., T. D. Goddard, …, T. E. Ferrin. 2021. UCSF ChimeraX: structure visualization for researchers, educators, and developers. *Protein Sci.* 30:70–82.

33. Meng, E. C., E. F. Pettersen, ..., T. E. Ferrin. 2006. Tools for integrated sequence-structure analysis with UCSF Chimera. *BMC Bioinf.* 7:339–410.

34. Tiwari, S. P., S. Chhabra, ..., O. Miyashita. 2020. Computational protocol for assessing the optimal pixel size to improve the accuracy of single-particle cryo-electron microscopy maps. *J. Chem. Inf. Model.* 60:2570–2580.

35. Wilkinson, M. E., A. Kumar, and A. Casañal. 2019. Methods for merging data sets in electron cryo-microscopy. *Acta Crystallogr. D Struct. Biol.* 75:782–791.

36. Sheng, Y., P. J. Harrison, ..., D. K. Clare. 2022. Application of super-resolution and correlative double sampling in cryo-electron microscopy. *Faraday Discuss.* 240:261–276.

37. Wang, Y.-F., R. Dutzler, ..., T. Schirmer. 1997. Channel specificity: structural basis for sugar discrimination and differential flux rates in maltoporin. *J. Mol. Biol.* 272:56–63.

38. Dutzler, R., Y. F. Wang, ..., T. Schirmer. 1996. Crystal structures of various maltooligosaccharides bound to maltoporin reveal a specific sugar translocation pathway. *Structure.* 4:127–134.

39. Pratt, L. R. 2002. Molecular theory of hydrophobic effects: "She is too mean to have her name repeated. *Annu. Rev. Phys. Chem.* 53:409–436.

40. Croll, T. I. 2018. ISOLDE: a physically realistic environment for model building into low-resolution electron-density maps. *Acta Crystallogr. D Struct. Biol.* 74:519–530.

41. Zhou, H., and Y. Zhou. 2002. Distance-scaled, finite ideal-gas reference state improves structure-derived potentials of mean force for structure selection and stability prediction. *Protein Sci.* 11:2714–2726.

42. McGreevy, R., A. Singharoy, ..., K. Schulten. 2014. xMDFF: molecular dynamics flexible fitting of low-resolution X-ray structures. *Acta Crystallogr. D Biol. Crystallogr.* 70:2344–2355.

43. Jumper, J., R. Evans, ..., D. Hassabis. 2021. Highly accurate protein structure prediction with AlphaFold. *Nature.* 596:583–589.

Biophysical Journal, Volume *122*

Supplemental information

Automated simulation-based membrane protein refinement into cryo-EM data

Linnea Yvonnesdotter, Urška Rovšnik, Christian Blau, Marie Lycksell, Rebecca Joy Howard, and Erik Lindahl

Automated simulation-based membrane-protein refinement into cryo-EM data

Linnea Yvonnesdotter[1], Urška Rovšnik[1], Christian Blau[1], Marie Lycksell[1], Rebecca J. Howard[1], and Erik Lindahl[1]

*Correspondence: erik.lindahl@scilifelab.se

SUPPORTING MATERIAL

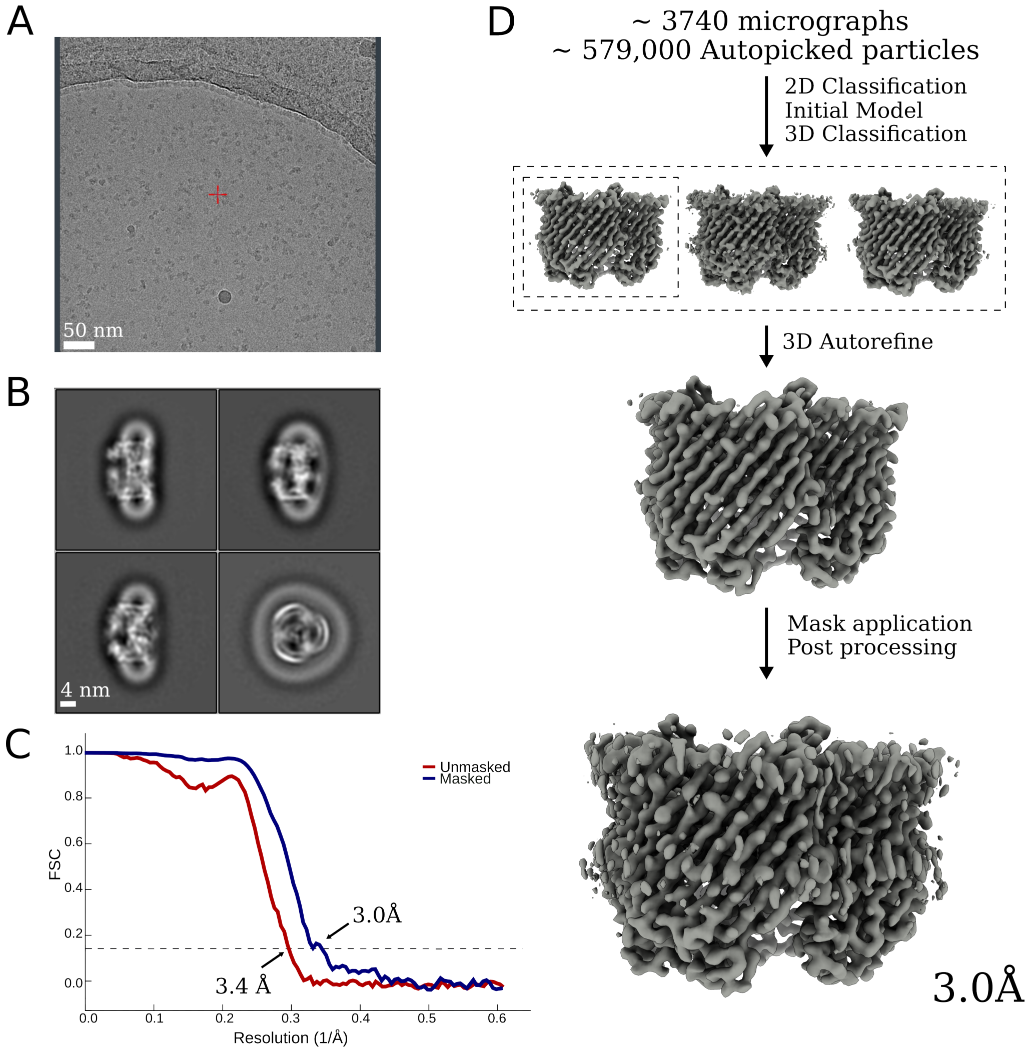

Figure S1: Maltoporin cryo-EM processing pipeline. **A** - Cryo-EM representative micrograph from the data collection. **B** - Representative 2D classes in multiple different orientations. **C** - FSC curves for unmasked (*red*) and masked (*blue*) map. **D** - Maltoporin processing pipeline with representative 3D classes, 3D refined density and a sharpened final map.

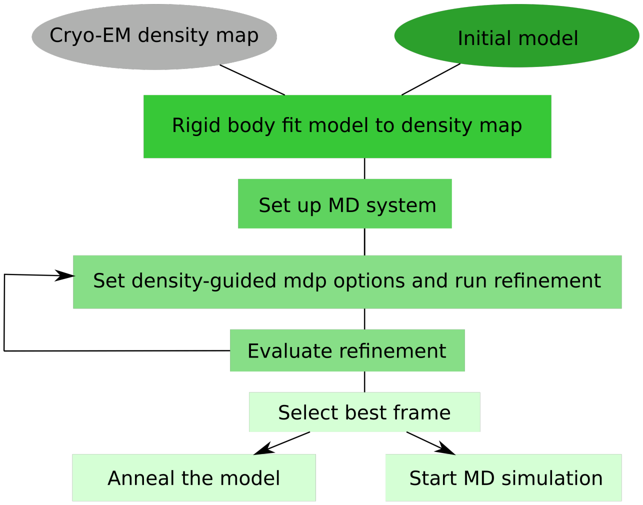

Figure S2: Suggested workflow for automated simulation-based refinement of a protein model to cryo-EM density.

Input - A cryo-EM density map or any 3D density map can be used as the target map. The initial model can for example be a model previously determined using an alternative technique or of an alternative conformation state, a homology model, etc.

Rigid body fit the model to the density map - An initial rough alignment of the initial model to the density aids in refinement. This can be done in ChimeraX using the Fit in Map tool (1). The fit protein model should then be saved relative to the target density map.

Set up MD system - The next step is to set up the molecular dynamics simulation system. For a simple solvated system GROMACS pdb2gmx, solvate, and genion functionalities can be used (2), followed by energy minimization. Membrane- or micelle-embedded systems can be set up using CHARMM-GUI bilayer builder (3) and CHARMM-GUI micelle builder respectively (4). We recommend that the size of the simulation system matches that of the target density to avoid inadvertent misalignment between map and model and checking the alignment between the target map and model, for example using VMD (5), as map and model coordinates can be interpreted differently by different tools.

Density-guided mdp options - Make sure to use the latest GROMACS version. Set the *gaussian-transform-spreading-width* according to the pixel size of the target density map as described in the methods section and more in depth by Blau et al. (6). The *density-guided-simulation-similarity-measure* affects the refinement behavior, as described by Blau et al. (6). Using relative-entropy might be advantageous for applications where a large-scale conformational change is expected or when starting from poor alignment. For refinement towards a density map containing density unaccounted for by the model relative-entropy is not recommended. We recommend *density-guided-simulation-adaptive-force-scaling* be set to true and the initial value of *density-guided-simulation-force-constant* to a low value (here we used $10e^1$ kJ/mol^{-1}). *density-guided-simulation-adaptive-force-scaling-time-constant* determines how frequently the fitting force is adapted. The default value of 4 ps has been successfully used both in this study and by Blau et al. (6), however, it might be advantageous to set the value higher for large-scale structural rearrangements, to allow for refinement during a longer period of time and thus sampling more of the conformational space.

Evaluate the refinement - We recommend that visual inspection, $FSC_{average}$, and GOAP-score be used as a minimal set of tools to evaluate the refinement. Here are some things to consider if the anticipated result has not been achieved. If a large conformational change is anticipated but not achieved it might be advantageous to refine using relative-entropy as the similarity measure, see more in (6). Another option is to increase the *density-guided-simulation-adaptive-force-scaling-time-constant*. A third option is to decrease the ruggedness of the target by fitting to a series of target maps (7). The ruggedness can be decreased by adding Gaussian noise or by downsampling the target maps. A tool for downsampling density maps is available at `https://gitlab.com/cblau/mrcsmooth/-/tree/main/`. If a part of the model is seemingly drawn outside of the density the target density map might have been extracted too narrowly. Due to the periodic nature of the simulation, it is possible that a part of the model close to the periodic boundary is refined toward the target density in an adjacent periodic box. This is more likely to happen using relative-entropy as the similarity measure as it has more long-ranged behavior, see (6). This can be solved by extracting a larger box around the density, changing the box size using the relion_image_handler tool (8), or changing the similarity measure.

Select the best frame - When satisfied with the refinement select the frame with the highest GOAP-score within the $FSC_{average}$ plateau region.

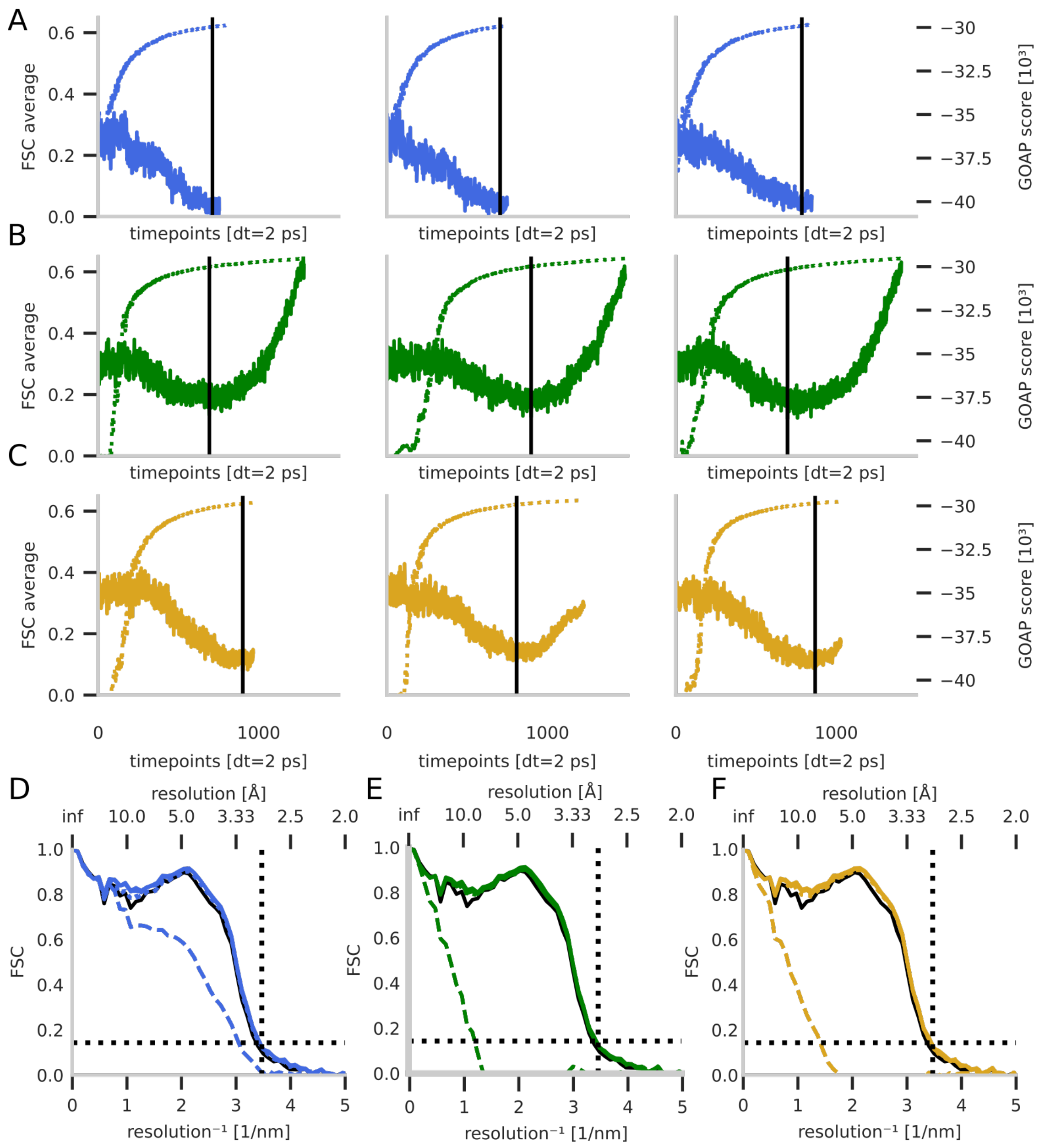

Figure S3: **A-C** - FSC$_{average}$ (dotted) and GOAP score (solid) over the refinement simulations (n=3) in solution (A), DDM (B) and POPC (C), best GOAP score frame marked with vertical black line. **D-F** - FSC of best fit position of density-guided simulations (n=3) of maltoporin (1MAL) in solution (blue), in DDM detergent micelle (green) and embedded in POPC (yellow) fit into cryo-EM density map, energy minimized models (dotted colored lines). Respective starting position marked in dashed lines and chimera fit-to-map rigid body fit starting model (solid black). Black dotted horizontal line at 0.143 and vertical at the lowest estimated local resolution of the map, 2.88 Å.

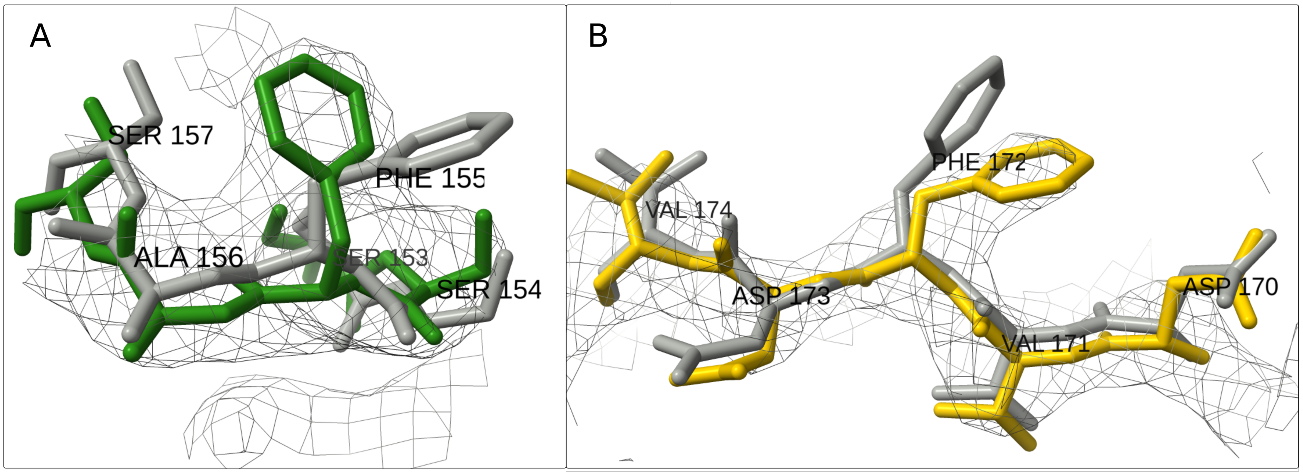

Figure S4: Local improvements in model fit to cryo-EM density after automated simulation-based refinement. **A** - Extracellular loop of maltoporin, showing the initial model (gray sticks) embedded in a DDM detergent micelle, equilibrated and rigid-body fit to the target cryo-EM density (gray mesh). Final model after density-guided simulations is shown in green. **B** - Transmembrane β-strand of maltoporin, showing initial model (gray sticks) embedded in a POPC bilayer, equilibrated and rigid-body fit to the density (gray mesh). Final model after density-guided simulations is shown in yellow.

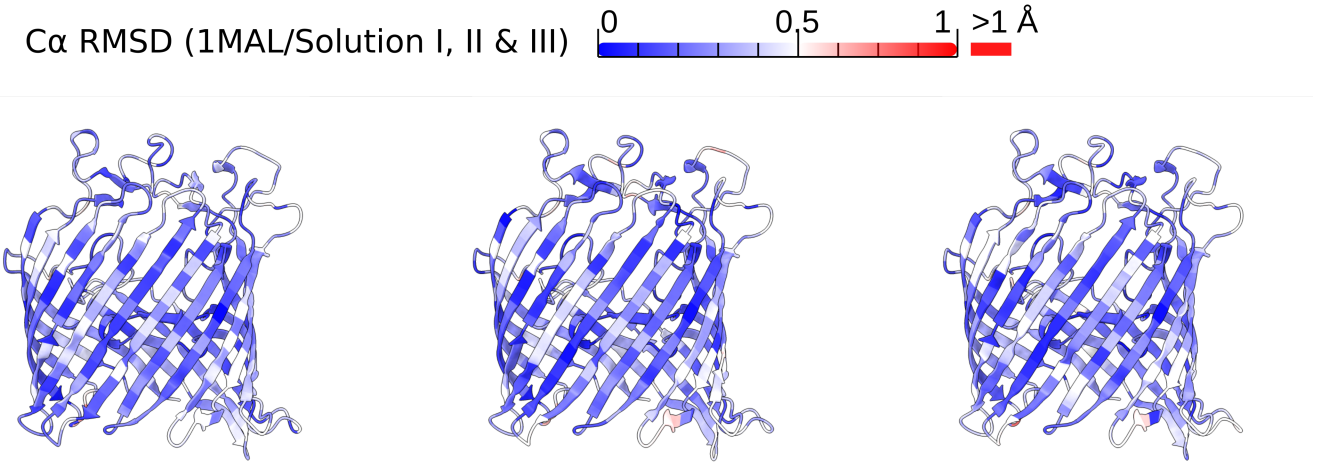

Figure S5: Moderate adjustments to secondary structure distributed throughout maltoporin after automated simulation-based refinement. Models (left—right) show final annealed state of chain A in three replicate fitting simulations (I–III) performed in solution, colored by Cα root mean-squared deviation from template X-ray structure (PDB ID 1MAL, blue=0 Å, red=1 Å).

REFERENCES

1. Pettersen, E. F., T. D. Goddard, C. C. Huang, E. C. Meng, G. S. Couch, T. I. Croll, J. H. Morris, and T. E. Ferrin, 2021. UCSF ChimeraX: Structure visualization for researchers, educators, and developers. *Protein Science* 30:70–82.

2. Páll, S., A. Zhmurov, P. Bauer, M. Abraham, M. Lundborg, A. Gray, B. Hess, and E. Lindahl, 2020. Heterogeneous parallelization and acceleration of molecular dynamics simulations in GROMACS. *The Journal of Chemical Physics* 153:134110.

3. Wu, E. L., X. Cheng, S. Jo, H. Rui, K. C. Song, E. M. Dávila-Contreras, Y. Qi, J. Lee, V. Monje-Galvan, R. M. Venable, et al., 2014. CHARMM-GUI membrane builder toward realistic biological membrane simulations.

4. Cheng, X., S. Jo, H. S. Lee, J. B. Klauda, and W. Im, 2013. CHARMM-GUI micelle builder for pure/mixed micelle and protein/micelle complex systems.

5. Humphrey, W., A. Dalke, and K. Schulten, 1996. VMD: visual molecular dynamics. *Journal of molecular graphics* 14:33–38.

6. Blau, C., L. Yvonnesdotter, and E. Lindahl, 2022. Gentle and fast all-atom model refinement to cryo-EM densities via Bayes' approach. *bioRxiv* `https://www.biorxiv.org/content/early/2022/09/30/2022.09.30.510249`.

7. McGreevy, R., A. Singharoy, Q. Li, J. Zhang, D. Xu, E. Perozo, and K. Schulten, 2014. xMDFF: molecular dynamics flexible fitting of low-resolution X-ray structures. *Acta Crystallographica Section D: Biological Crystallography* 70:2344–2355.

8. Nakane, T., A. Kotecha, A. Sente, G. McMullan, S. Masiulis, P. M. G. E. Brown, I. T. Grigoras, L. Malinauskaite, T. Malinauskas, J. Miehling, T. Uchański, L. Yu, D. Karia, E. V. Pechnikova, E. de Jong, J. Keizer, M. Bischoff, J. McCormack, P. Tiemeijer, S. W. Hardwick, D. Y. Chirgadze, G. Murshudov, A. R. Aricescu, and S. H. W. Scheres, 2020. Single-particle cryo-EM at atomic resolution. *Nature* 587:152 – 156.
